# Direct Correlation Between Ligand-Induced α-Synuclein Oligomers and Amyloid-like Fibril Growth

**DOI:** 10.1038/srep10422

**Published:** 2015-05-28

**Authors:** Martin Nors Perdersen, Vito Foderà, Istvan Horvath, Andreas van Maarschalkerweerd, Katrine Nørgaard Toft, Christoph Weise, Fredrik Almqvist, Magnus Wolf-Watz, Pernilla Wittung-Stafshede, Bente Vestergaard

**Affiliations:** 1 Department of Drug Design and Pharmacology, University of Copenhagen Universitetsparken 2, DK-2100 Copenhagen, Denmark; 2 Department of Chemistry, Umeå University, S-901 87 Umeå, Sweden; 3 Umeå Centre for Microbial Research, Umeå University, 901 87 Umeå Sweden

## Abstract

Aggregation of proteins into amyloid deposits is the hallmark of several neurodegenerative diseases such as Alzheimer’s and Parkinson’s disease. The suggestion that intermediate oligomeric species may be cytotoxic has led to intensified investigations of pre-fibrillar oligomers, which are complicated by their transient nature and low population. Here we investigate alpha-synuclein oligomers, enriched by a 2-pyridone molecule (FN075), and the conversion of oligomers into fibrils. As probed by leakage assays, the FN075 induced oligomers potently disrupt vesicles *in vitro,* suggesting a potential link to disease related degenerative activity. Fibrils formed in the presence and absence of FN075 are indistinguishable on microscopic and macroscopic levels. Using small angle X-ray scattering, we reveal that FN075 induced oligomers are similar, but not identical, to oligomers previously observed during alpha-synuclein fibrillation. Since the levels of FN075 induced oligomers correlate with the amounts of fibrils among different FN075:protein ratios, the oligomers appear to be on-pathway and modeling supports an ‘oligomer stacking model’ for alpha-synuclein fibril elongation.

Amyloid deposits of protein fibrils are the hallmarks of several grave diseases, one example being Lewy bodies in Parkinson’s disease^1^. Protein fibrils are elongated rod-like structures with a length up to several μm, and their formation takes place both *in vivo* and *in vitro* through several interconnected mechanisms^2^. The development of strategies to control and eventually reverse the process crucially depends on information about how fibrils are formed and the molecular basis of any disease-related mechanism of cytotoxicity.

Alpha-synuclein (aSN) is a 140-amino acid intrinsically disordered protein. It is a main constituent of the Lewy bodies (LB) which can be found in the brains of patients affected by Parkinson’s Disease and other neurodegenerative diseases^1^. LBs were originally thought to be causative of disease progression, but the amount of LB found in the patient´s brain does not correlate directly with neurodegeneration^3^. On the contrary, there are increasing evidences of the cytotoxic effect induced by transiently formed soluble oligomeric species^4^,^5^,^6^,^7^ and of oligomers exacerbating cytotoxicity of fibrils^8^. It is however not known whether such harmful oligomers are on- or off-pathway in the fibrillation pathway. Consequently, it is also not known whether therapeutically induced clearance of fibrils would, non-deliberately, worsen the cytotoxic effect by returning the protein from the fibril state to the oligomeric state. In the quest for efficient pharmaco-therapeutic strategies, it is thus of particular importance to evaluate the role of intermediate oligomers in the formation of the final fibrils, and to acquire a quantitative understanding of both structure and properties of such oligomers. Such tasks are challenging mainly because of the transient nature of such intermediate species and the need for using non-invasive methods of analysis, in order to not disturb potential equilibriums involved in their formation.

To control the highly dynamic nature of oligomers, a number of strategies for increasing their stability has been proposed, using mainly small-molecule substances, e.g. stimulants such as nicotine^9^, or neurotransmitters such as dopamine^10^ and serotonin^11^. Other approaches to stabilize the oligomers have included *de novo* design of small molecule compounds reminiscent of peptide structures^12^. One of these is the 2-pyridone compound FN075^13^. This compound causes rapid accumulation of intermediate aSN oligomeric species and increases the speed of aSN fibrillation^12^. Since FN075 induced oligomers could seed fresh aSN aggregation reactions^14^ it was proposed that the FN075-induced oligomers were on-pathway towards fibrils^12^,^14^. Using a Small Angle X-ray Scattering (SAXS) based approach, the low resolution structure of an aSN oligomeric species in absence of any small molecule was previously resolved^15^. These aSN oligomers were also suggested to be on path towards fibrils, the latter proposed to form via an “oligomer stacking” mechanism^15^. Various other aSN oligomers have been isolated, for example using mechanical stress for their formation^16^. These aSN oligomers are stable after purification, which suggests that the oligomers are off-pathway in the fibril formation process, and furthermore seem to inhibit fibrillation^16^. These results highlight how subtle changes in sample preparation and/or oligomer isolation can lead to changes in the structures and properties of the oligomers and poses a serious challenge for the experimental analysis of such highly equilibrium-dependent species. Further studies are therefore strongly needed to assess the role of the various oligomers related to the fibrillation pathway. Moreover, understanding and rationalizing the role of small molecules such as FN075 during the fibrillation pathway represents one of the possible routes for the development of strategies to reverse/inhibit the self-assembly process, being of crucial importance in medical and biopharmaceutical sciences.

Here we combine the ability of the small molecule FN075 to trigger aSN oligomerization with the possibility of obtaining low-resolution structural information in solution via SAXS data analysis. Importantly, our analysis does not require prior isolation of the oligomeric species, and SAXS is a non-invasive method. We present the low resolution structure of the FN075 induced aSN oligomer and demonstrate that these oligomers disrupt vesicles with higher potency than either fully matured fibrils or intermediate fibrillating samples (i.e. in the absence of FN075 but containing monomers, intermediate oligomers and fibrils). Such high perturbing activity on a lipid bilayer could suggest that such an intermediate oligomeric species could be cytotoxic, if produced *in vivo*. We show how the levels of oligomers are correlated with the levels of fibrils at different FN075 concentrations, which suggests that the FN075 oligomers are embedded within the final fibrils (i.e. on-pathway oligomers). Furthermore, we compare the aSN oligomer and fibril structures formed with and without FN075. We reveal that oligomers formed with and without FN075 are similar, although not identical, and that fibrils formed under the two conditions are morphologically and microscopically indistinguishable. Finally, we show, that the fibril shape can be reproduced, by the stacking of oligomers. Under the experimental conditions investigated, this supports a fibril elongation model including a vesicle-disrupting on-pathway oligomer.

## Results and Discussion

### FN075-induced structural conversion of aSN

**Figure 1:**
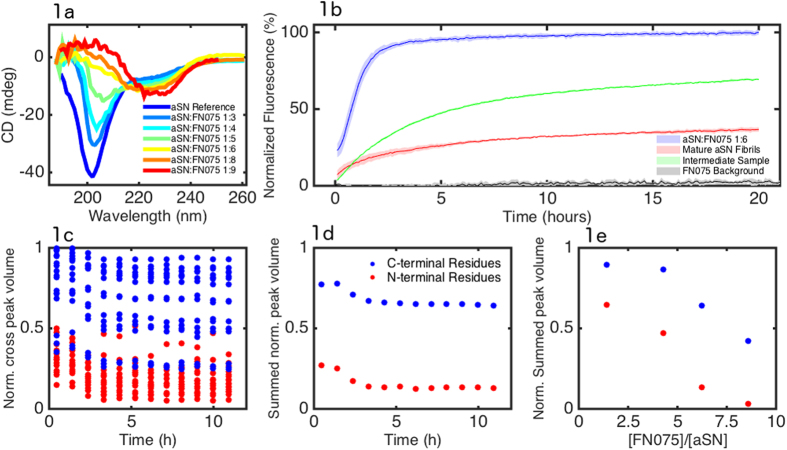
Characterization of the structural conversion induced by FN075. **a**) CD spectra recorded with increasing amounts of FN075. Color codes are defined in the legend, specifying aSN:FN075 ratios from 1:0 to 1:9. **b**) Calcein Release assay from DOPG vesicles from oligomers induced by FN075 (blue) compared to the release by fully mature fibrils (red), and an intermediate sample (green), the latter two samples not containing any FN075. The line is the average (n = 3) and the shaded area corresponds to the standard deviation.. The data is normalized by complete leakage, which is reached by the addition of the detergent Triton X-100. The negative control (grey line) is made with addition of buffer containing FN075, but no protein. **c**) Residue specific attenuation of the HSQC cross-peaks at a 6:1 aSN:FN075 ratio; **d**) Summed NAC/N-terminal residue attenuations (blue) and summed C-terminal residue attenuations (red); **e**) Final summed NAC/N-terminal residue (blue) and C-terminal (red) attenuations as a function of aSN:FN075 ratio.

In order to investigate the structural characteristics of the FN075-induced oligomer we optimized the experimental conditions for the oligomer formation. Far-UV CD was recorded for native aSN and for samples with different aSN:FN075 ratios with a maximum of 1:9 (Fig. 1a).

From the data it is clear that the minimum around 202 nm, characteristic of unstructured proteins, becomes less pronounced with increasing concentration of FN075. Samples with higher aSN:FN075 ratios present a more pronounced minimum around 220–230 nm and a maximum in the region 188-208 nm. These two features stem from the gradual conversion from random coil to β-sheet structure induced by FN075^17^. Moreover, dynamic light scattering (DLS) experiments show that aSN samples without FN075 are characterized by a mono-modal distribution of the hydrodynamic diameter around 6–8 nm, while the hydrodynamic diameter is centered around 30 nm for the FN075 induced oligomers^14^. Four different aSN:FN075 ratios were also investigated by the NMR approach used previously by us (Fig. 1c)^12^. In brief, attenuation of the N-terminal peaks without changes in chemical shifts indicated formation of aSN oligomers^12^. We found that oligomerization was almost complete at a molar ratio of 1:6 aSN:FN075 and that the oligomers appeared stable for the duration of the experiments. These results, in concert with the CD results and the increase in size detected by dynamic light scattering, show that when adding FN075 to aSN samples, a formation of β-strand rich oligomers takes place with an almost complete conversion to an oligomeric state at an aSN:FN075 ratio of 1:6. Since there was no chemical shift changes observed for the monomer spectrum upon FN075 addition, it shows that the remaining monomers did not change their structure in the presence of FN075 and the oligomers (please cf. supplementary information for a more detailed description).

### The induced oligomers disrupt lipid vesicles

Previous studies have suggested that oligomeric aSN species are essential to cytotoxic protein:lipid interactions, based on the observation that they disrupt anionic lipid vesicles with higher potency than fully matured fibrils or monomeric species^15^,^16^. We therefore investigated if the oligomers induced by FN075 also have the ability to disrupt vesicles. The results are shown in Fig. 1b, where it is clear that FN075 oligomers disrupt vesicles both efficiently and rapidly. The release is compared to that induced by fully matured fibrils and to that caused by the addition of an intermediate sample containing a mixture of monomers, oligomers and fibrils^18^, neither of the latter two samples containing FN075. Both fully fibrillated aSN and the intermediate samples disrupt vesicles at much reduced rate compared to FN075-oligomers. The half maximum is reached after 46 min for the oligomer solution, whereas it takes 1 h43 min and 2 h48 min for fibrils and early species respectively. Furthermore, the samples with fibrils or early species both reach a steady state below 100% as previously reported^18^, in contrast to the FN075 oligomer sample which gives 100% release.

From Fig. 1b we also see that fully matured fibrils have lower potency than the intermediate sample. From previous studies we know that monomers also have lower potency^15^, strongly suggesting that the increase in potency of the intermediate sample is due to the presence of oligomers. This is in clear agreement with the even further increased potency of the FN075 induced oligomers.

### Effect of FN075 on the amyloid fibril morphology

**Figure 2:**
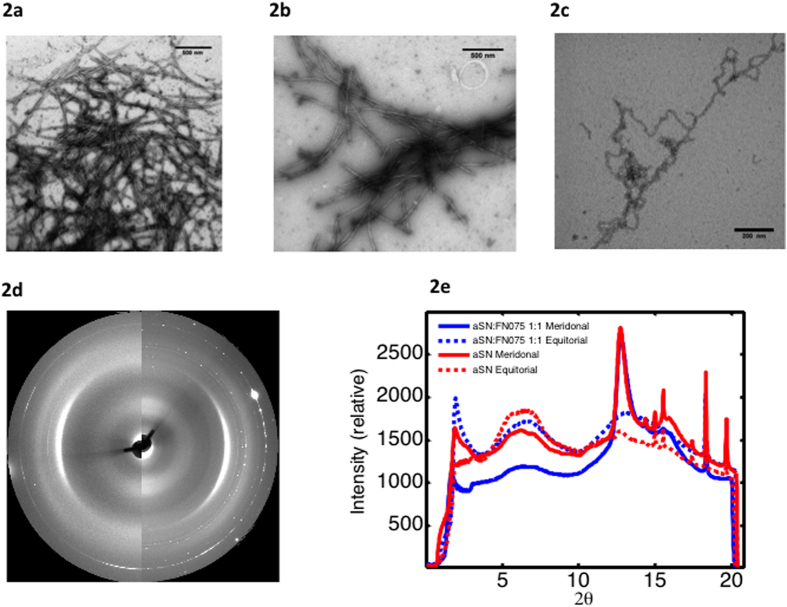
Characterization of the fibrils using TEM and fiber diffraction. **a**–**c**) aSN fibrils prepared **a**) with agitation in the absence and **b**) presence of FN075 in comparison with **c**) fibrils prepared without agitation but in the presence of FN075. **d**) the 2D FD images from fibrils formed in the presence (left half circle) and absence (right half-circle) of FN075. **e**) Superimposed and radially averaged FD data. Meridional and equatorial data are shown with solid and dotted lines respectively, while data from fibrils with and without FN075 are plotted in blue and red respectively.

We performed TEM imaging from samples produced by the standard fibrillation protocol for aSN (i.e. in a plate-reader by applying agitation in the presence of a glass bead) (Fig. 1a). We compared this to fibrils produced by the same protocol, but including FN075 (molar ratio aSN:FN 1:2) (Fig. 1b) and reveal that these fibrils are morphologically very similar. TEM also revealed that the fibrils formed in the presence of FN075 but without applying agitation (Fig. 2c) exhibit more twists and turns. We attribute this difference to the agitation used in the platereader assay, which is known to cause fibril fragmentation^19^,^20^. The fragmentation has reduced the fibril curvature, making lateral association easier, which can also be seen in the images. The individual fibrils, however, appear to have the same cross sectional dimensions regardless if they have been incubated in a platereader or in a test-tube (see supplementary information Figure S1). Finally it is evident from the micrographs that smaller aggregates co-exist with the fibrils.

Considering the similarity between standard fibrils and those prepared in the presence of FN075, we employed fiber diffraction (FD) to investigate if the microscopic internal structure of the fibrils remains the same. Samples containing 1:1 and 1:2 aSN:FN075 as well as aSN reference fibrils were investigated. Both reference aSN fibrils (Fig. 2d left) and fibrils prepared in the presence of FN075 (Fig. 2d right) show meridional (4.7 Å) and equatorial reflections (broad reflection around 10 Å) typical for the cross-β pattern (Fig. 2d-e) and at distances in agreement with previously published values^21^. There is thus no indication that FN075 induces any microscopic structural changes in the fibrils, supporting the conclusion from TEM that the fibrils formed in presence of FN075 are similar to normal fibrils.

### FN075 oligomers co-exist with fibrils and monomers

**Figure 3:**
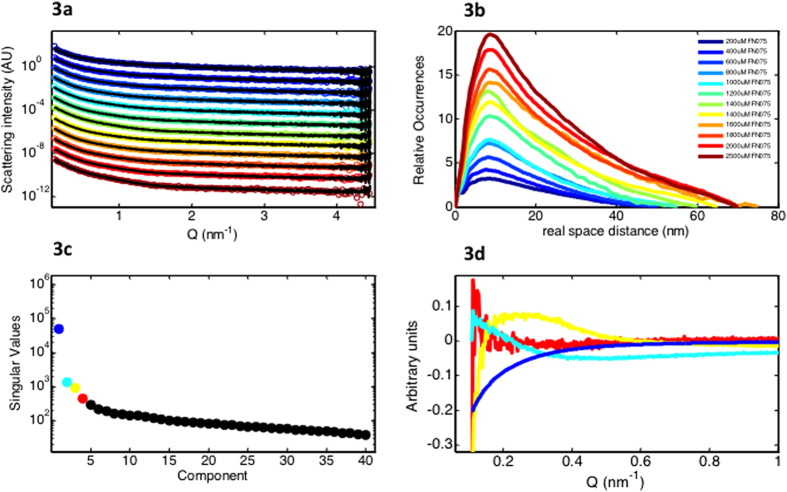
SAXS data from titration series with aSN and FN075. **a**) 350 μM aSN with increasing amounts of FN075 (colors are defined in legend) with the fit from the decomposition (black). Only one measurement for each concentration is shown, but a time series including five time-points was performed for the first eight samples while three time-points were recorded for the highest concentrations giving a total of 46 measurements on aSN:FN075 samples (see supplementary information Figure S2). Curves are offset by an 10^k^, where k is the curve number, for viewing purposes. **b**) The pair distance distribution from the samples in 3**a**. **c**) Singular values from the singular value decomposition. The four largest singular values are plotted in blue, turquoise, yellow and red. **d**) The left singular vectors from the singular value decomposition. The coloring corresponds to the colors in 3**c**.

From previous studies^12^, we know that the lag-time of fibrillation is decreased in the presence of FN075. Although our data demonstrate that the presence of FN075 does not affect the final fibril core structures, it is not clear whether aSN follows the same fibrillation pathway in the absence or presence of FN075. In order to follow the structures involved in aSN fibril formation with FN075, we applied SAXS. A wide range of aSN:FN075 ratios was investigated and Fig. 3a shows representative scattering curves from the titration series, which consist of a total of 42 scattering curves from samples prepared by addition of FN075 to a protein stock. *Gnom* from the ATSAS suite was used for the indirect Fourier transformation, which yields the pair distance distribution (p(r)-function), that describes the relative occurrences of intra-particle distances^22^. The p(r)-functions from the data (Fig. 3b) show an increasing peak at the lower real space distances, with a slow linear decline towards the maximal dimension, typical for elongated particles. This reveals increasing fibrillation in the presence of increasing amounts of FN075, as also indicated by radius of gyration, forward scattering and the maximal dimension derived from the sample data (Table 1).

Our NMR data have shown that our samples consisted of mixtures of oligomers and monomers, while TEM showed fibrils and small sized aggregates. Together, the data suggest that at least three species exist during the FN075 induced aSN fibrillation process (monomers, oligomers and fibils). The minimum number of species in solution can also be determined by singular value decomposition (SVD) of the SAXS data. Figure 3c shows the result, with a very dominant first component. The singular values for the second and third component are substantially smaller than the first component, but still significant. Fig. 3d shows the left singular vectors for the first four components. The first three components exhibit clear features while the fourth resembles random noise, strongly suggesting that the number of species in solution that significantly contribute to the data series is three.

**Figure 4:**
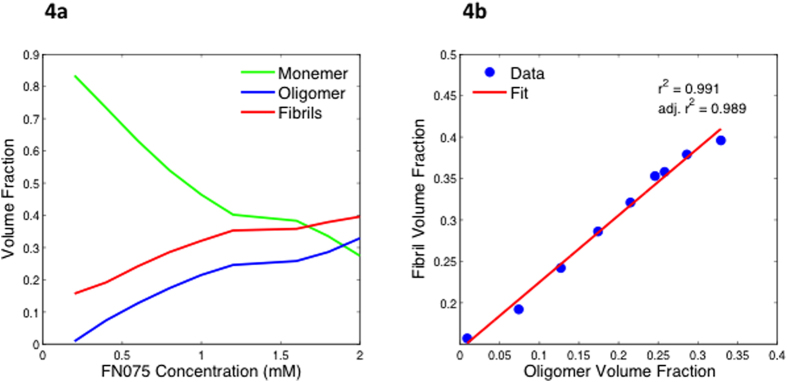
Volume fractions from the decomposition of the titration series. **a**) Plot of the volume fractions as function of FN075 concentration, revealing the development in solution of monomers (green), fibrils (red) and oligomers (blue). Notice the constant relationship between the amount of fibrils and oligomers throughout the series. A similar investigation of the development over time while keeping the ratio constant is included in supplementary information. **b**) Linear regression of the fibril volume fraction as a function of oligomer fraction. Notice the high correlation coefficient.

Due to the additive nature of X-ray scattering, the SAXS signal from the FN075 oligomer could be isolated using the program *Oligomer* from the ATSAS suite^22^ and the approach used previously for other fibrillation processes^15^,^23^. The decomposition rests on accurate determination of the number of species in solution, which we have determined to be three. From our complementary analysis (NMR, TEM, FD, DLS), the three species are assigned to monomer, FN075 induced oligomer and fibril. Representative fits from the decomposition of the SAXS data are shown together with the raw data in Fig. 3a. It can be seen that using the same three components, monomer, oligomer and fibril, the entire range of curves could be satisfactorily fitted within the entire data range collected. From the decomposition, the volume fractions of the three species can be estimated (Fig. 4a).

These data reveal a clear relation between the amount of oligomers and fibrils formed at each FN075 condition. Specifically, with higher FN075-to-protein ratio, the volume fraction of oligomers increases as do the fibril volume fraction. The strong correlation between oligomer and fibril volume fractions is highlighted in Fig. 4b, where we show that the amount of fibrils can be predicted directly from the volume fraction of oligomers. This, taken together with the fact that i) when FN075 is added to aSN both oligomers and fibrils become populated and ii) the presence of FN075 increases aSN fibrillation rate^12^, strongly indicates that the oligomers participate in the formation of fibrils (i.e. they are on-pathway). If this was not the case, induction of oligomers should slow the fibrillation down. It is furthermore interesting that the ratio between oligomer and fibril volume fraction does not remain constant. Specifically, from Fig. 4a is evident that oligomer:fibril ratio increases at higher FN075 concentration. This means that the equilibriums are shifted towards the oligomer as the amount of FN075 is increased. This suggests that the oligomers become increasingly stable with increasing amount of FN075 bound. The implication of this is that different solution states can be prepared using different molar ratios of FN075 and aSN. In the low ratio regime, the oligomers are still relatively unstable and will hence proceed to fibrillate to a higher degree, and the degree of fibrillation will correspondingly decrease, as the oligomers get more and more stable. Importantly, the FN075 titrated samples were stable throughout the time frame of the experiments (see supplementary information Figure S2), reducing the transient nature of the oligomer. This is a notable difference from the experimental conditions investigated previously^15^. There, the oligomers were not stable and hence proceeded to dissociate or fibrillate completely, restricting the time frame and types of experiments that could be carried out. Here, the oligomers can be induced and the sample will be stable for at least 24 h.

### Deduction of the stacking mode

**Figure 5:**
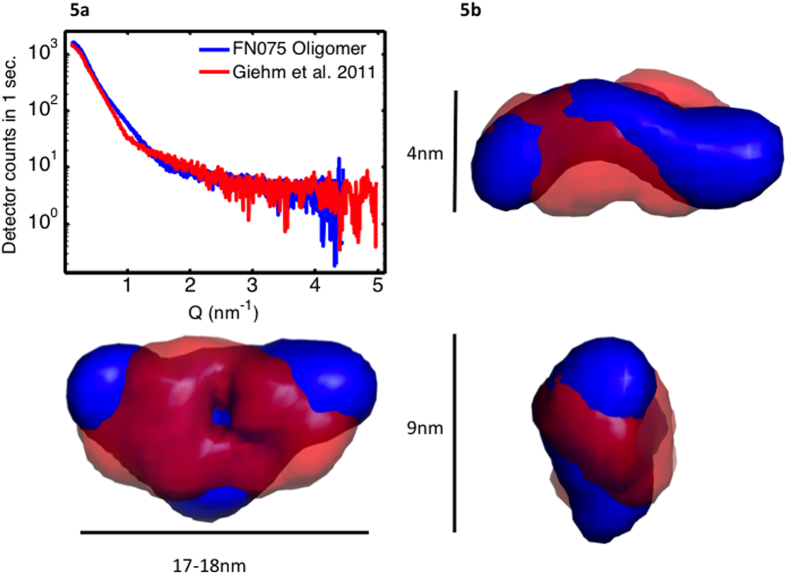
The isolated oligomer data from the decomposition and the low resolution structure obtained from it. **a**) The isolated data from FN075 induced oligomer formation (blue) compared to that isolated from native aSN fibrillation (red). **b**) *Ab initio* SAXS models of oligomers formed in the presence (blue) and absence (red) of FN075. The most representative model from the ensemble is shown. The three views are rotated 90°.

Based on the suggestion that the fibrils are formed from oligomers, we wanted to investigate if that elongation could happen via oligomer stacking. For that we used the *DAMMIN* algorithm^24^ to model the low resolution structure of both the oligomer and the fibrils. For the oligomer, we used the data obtained from the decomposition. Already from the raw isolated data curve, it is clear that the decomposed aSN:FN075 oligomer data are different from the oligomer data that we have reported earlier and obtained in absence of FN075 (Fig. 5a).

Figure 5b shows the low-resolution structure for the FN075 oligomer (in blue), together with the aSN oligomer model that we have published previously (in red). The models are shown from three views (front view, side view and top view) to better visualize the structural features. The volume of the FN075 oligomer corresponds to a molecular mass of 220 kDa, or 15 aSN protein molecules. It is clear that the FN075 oligomer (blue) does not differ substantially in volume or apparent mass from the oligomer observed in the absence of a small molecule compound. However, compared to the structure we reported previously, the model of the FN075 induced oligomer is slightly more extended and with a pronounced central bend. This suggests that the FN075 oligomer could represent a modified fold of the native wreath-shaped oligomer^15^ and hence bear significant similarities, also microscopically, to the previously published model. Interestingly, it has been reported that the FN075 induced oligomer immediately dissociates upon dilution during gel filtration^12^. This is fully in accordance with our suggestion, that the FN075 induced oligomer exists in equilibrium with the native monomer and the aSN fibrils. That is, FN075 is an on-pathway oligomer, and its concentration is dependent on the presence of native monomer and aSN fibrils, and regulated by the overall concentration of FN075. This is in contrast to other oligomers reported^9^,^10^,^11^,^16^, which highlights the highly sensitive nature of the aggregation prone, transient species and how different methods/protocols will potentially reveal different aspects of the oligomerisation processes happening during fibrillation^25^. Each such species may reveal complementary and equally important aspects, which are relevant for an improved understanding of aSN fibrillation.

**Figure 6:**
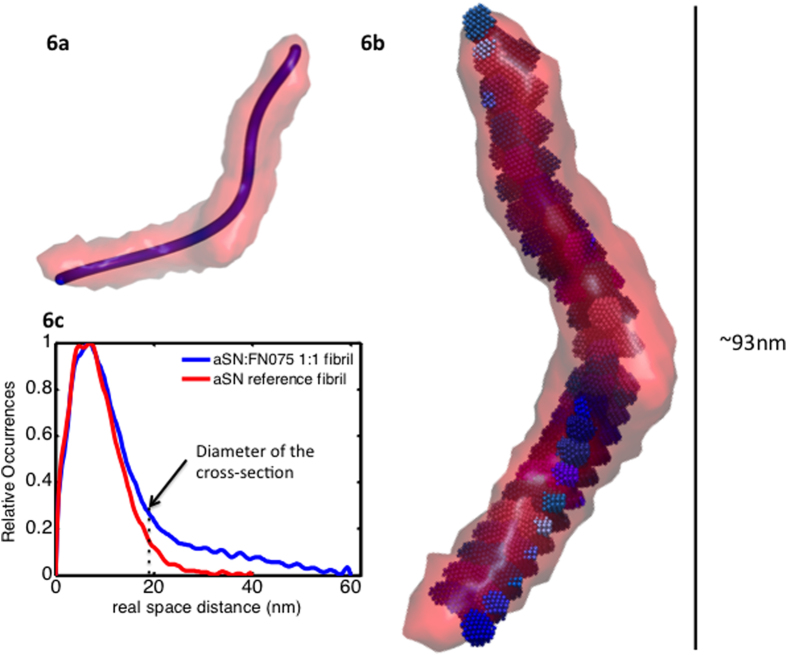
Analysis of the FN075 induced fibrils and proposed stacking pattern. **a**) The filtered low resolution SAXS model of the repeating unit of fibrils produced in presence of FN075, shown with the parameterized trace calculated in Matlab. **b**) The low resolution oligomer model stacked inside the repeating fibril unit according to the trend shown in 6**a**. **c**) The cross sectional p(r)-functions for standard fibrils (red) and fibrils produced in presence of FN075 (blue). The two fibrils have comparable cross-sectional dimensions, and a diameter in accordance with the width of the oligomeric species. A stabled line highlights the latter.

Fibrils are too large to be modeled in their entire length, but the repeating unit of the fibril can be investigated^23^. From this, important insights on how the oligomers can be embedded within a fibril can be obtained. For rod-like structures, it is possible to calculate the p(r)-function of the cross-section. We have done this for fibrils produced in the presence and absence of FN075 (blue and red in Fig. 6c, respectively), and it is clear that the cross-sectional dimensions are comparable for the two species. The tails in the cross-sectional p(r)-function are artifacts stemming from the curvature of the fibrils, which makes them imperfect rods. Still, it is possible to estimate the diameter of cross-section, which is highlighted by a stabled line in 6c. This estimate is in good agreement with the length of the FN075 oligomer. The repeating unit is ~210 Å wide and ~930 Å long (Figs. 6a-6b). The longest dimension in the model shown is approximately 530 Å.

For the stacking analysis, the filtered models were used and the stacking was rationalized and performed in Matlab (see Methods for details). To fit the oligomer model into the fibril model, the fibril model was parameterized with respect to the Z-direction using polynomials. This allows us to describe the translation of oligomers in an objective and systematic manner. The trajectory that resulted from the parameterization can be seen in Fig. 6a (blue line). The equations were then used to translate the oligomers along the fibril unit (Fig. 6b). The significant shape of the oligomer suggests that consecutive oligomers should be rotated around the Y-axis by 90° which is done in the final model depicted in Fig. 6. A total of 22-23 oligomers could be fitted into the fibril model. This is slightly less than what we observed previously (26 oligomers in a span of 900 Å, compared to 23 oligomers in a span of 1100 Å), which can be explained by the increased rotation of the oligomers.

It remains of obvious interest to consider, whether the FN075 stabilized oligomer, which seems to stack to elongate aSN fibrils formed in the presence of FN075, reveals an elongation principle which also is relevant for native aSN fibrillation principles. Although such a conclusion cannot be made with certainty, several observations suggest that this is the case. Firstly, the mature fibrils have comparable morphology and microscopic structure in the absence and presence of FN075. Secondly, far-UV CD suggests that the oligomers formed with and without FN075 have similar contents of secondary structure elements. Thirdly, although the FN075 oligomer is not identical to the native oligomer, the similarities in terms of volume and overall dimensions, and the wreath-shaped appearance, are striking. We suggest that the FN075 induced oligomer is a modulated form of the native oligomer, where binding of the small-molecule induces only subtle structural changes but the principal architecture of the two oligomers is the same.

## Materials and Methods

### Expression and Purification

aSN was expressed in *E. coli* BL21(DE3) cells. The aSN construct was a kind gift from Bioneer, Hørsholm, Denmark. Purification was done as described by Huang and co-workers^26^, with two modifications: 1) the supernatant from the osmotic shock treatment, containing aSN was boiled before ion-exchange chromatography and 2) fractions containing aSN from the ion-exchange chromatography was further purified using gelfiltration with a superdex 200 pg HiLoad 16/600 column. Fractions containing aSN without contaminations were identified using SDS-PAGE. These fractions were pooled and dialyzed against MQ water over 48 h. Finally, the dialyzed solution was lyophilized using a Heto LyoPro 6000.

### Protein stock preparations

Protein was dissolved in 20 mM phosphate and 150 mM NaCl buffer. Samples were transferred to 0.22 μm centrifugal filter (MilliPore), and filtered at 16,162 *g*. Concentration was then determined on a Nanodrop (Thermo Scientific) using the extinction coefficient of 0.412 mg^−1^ cm^3^. FN075 was added from a 100 mM stock in DMSO as described previously^12^.

### Amyloid fibril formation by platereader assay

Fibrillation was carried out in an Optima Fluostar platereader. Temperature was set to 37 °C. A glass bead with a diameter between 3.0 mm and 3.2 mm was added to each well and the platereader was programmed to shake for 280 s in between measurements. The shaking was orbital with 300 rpm and the amplitude was 2 mm. Thioflavin T (ThT) fluorescence (20 μM) was used to follow the fibrillation process (λ_ex_ = 450 nm, λ_em_ = 480 nm).

### Circular Dichroism

Circular Dichroism (CD) was recorded on an Olis CD module. The protein concentration was 21 uM and the cuvette length 1 mm. The temperature control was set 20 °C and integration time set to increase as a function of high tension voltage. Typically, 5 acquisitions were acquired for each sample.

### Preparation of Liposomes

Small unilamellar vesiscles (SUV) containing calcein in self-quenching concentrations were prepared to test the ability of FN075 oligomers to disrupt anionic lipid bilayers. POPG (1-palmitoyl-2-oleoyl-*sn*-glycero-3-phospho-(1’-*rac*-glycerol) [Avanti Polar Lipids] dissolved in chloroform was dried on a rotary evaporator and stripped 3 times with ethanol before drying overnight in round flasks to form lipid film. The lipid film was hydrated with 20 mM HEPES buffer (220 mM KCl, 0.06 mMCaCl_2_, 0.02 mM EDTA, 20 mM Calcein, pH 7.4) and the lipid solution was subsequently sonicated and whirled thoroughly to form multilamellar liposomes. SUV’s were obtained by extruding the liposomes 10 times through a double layer of 100 nm polycarbonate filter-membranes (Whatman) using a Lipex extruder (Nothern Lipids Inc.). Excess calcein was removed by gel-filtration in PD-10 columns (GE Healthcare) packed with Sephadex-50 (Sigma Aldrich). An average hydrodynamic diameter of approximately 100 nm and a narrow size distribution (polydispersity index below 0.1) was confirmed using a Malvern NanoZS (Malvern Instruments) equipped with a 633 nm laser and 173° optics.

### Calcein Release Assay

Dye release assays were performed in 96 well optical bottom plates (Thermo Scientific) on a Fluostar Optima plate-reader (BMG labtech). The ability of FN075 oligomers to induce calcein release was tested along with a fully fibrillated aSN sample and an intermediate sample containing native oligomers in equilibrium with native aSN and mature fibrils^18^. Measurements were performed in triplicate (lipid-protein molar ratio of 20:1) at 28 °C with excitation at 485 ± 5 nm and recording the calcein fluorescence emission at 520 ± 5 nm. Data points were recorded every 4 min. The measurement cycles included 5 s of orbital shaking (1 mm, 600 rpm) before each measurement. The measurements were terminated by addition of Triton-X (Sigma Aldrich) to obtain full release. The intrinsic stability of SUV’s after addition of equivalent amounts of FN075 in DMSO was confirmed to ensure that neither the ligand itself nor DMSO disrupted liposomes.

### Small Angle X ray Scattering

Samples for a titration series with increasing concentrations of FN075 were prepared as described above. Protein concentration in the entire titration series was 5 mg/ml, in a buffer consisting of 20 mM phosphate and 150 mM NaCl (PBS). FN075 was used in the range of 200 μM to 2500 μM which corresponds to 0.2% v/v to 2.5% v/v of DMSO. The effect of DMSO on aSN was assessed by a separate measurement where DMSO without FN075 was added to aSN. For optimal background subtraction, a concentration series with FN075 ranging from 0–500 μM in buffer, devoid of protein, was measured. As FN075 did not give rise to any signal, a buffer consisting of PBS with the appropriate amount of DMSO was used as background. See supplementary for details on the data collection and background subtraction.

Primary data analysis was carried out using *Primus* from the ATSAS suite^22^ and *ScÅtter*^27^. *Gnom* from the ATSAS suite was used to obtain the Pair Distance Distribution function^28^. Singular value decomposition (SVD) was carried out in Matlab. The data decomposition was performed using the program *oligomer* from the ATSAS suite^22^. Briefly, experimental data from the native species and a fully fibrillated sample were used as form factors together with the oligomer data isolated by us previously. These three form factors were fitted, using a linear combination, to all the data curves from the titration series. Residuals from the fits were inspected afterward and, if clear systematic trends were observed throughout the titration series, residuals were added to the oligomer form factor. This was done iteratively until a reliable fit was obtained. The coefficients from the linear fit, referred to as volume fractions, were also analyzed.

Dammin was used to obtain low resolution models for the oligomer and the repeating unit in the fibril^24^, see supporting information for details. For stacking of the oligomer inside the fibril, the filtered models from DAMAVER were used^29^. The filtered model is not fitted to the data and is instead based an average of all features in the ensemble, which is filtered down to the correct volume. The filtering is done by the occurrences of the feature so that the filtered model contains all the most typical features. By using the filtered model, we account for all the typical features in both the fibril and oligomer model ensemble. To stack the oligomers, the filtered models were loaded into Matlab and centered. The statistics toolbox was used to parameterize the fibril model in the Y and X dimensions with respect to the Z-direction, using polynomials. These equations were subsequently used to translate the oligomer model throughout the fibril shape hence enabling the development of an objective and optimized model for the fibril elongation by oligomer stacking. The bioinformatics toolbox was used to write the new coordinates to protein data bank (pdb) files.

### Transmission Electron Microscopy (TEM)

To investigate fibrils formed by sole addition of FN075, an aSN stock was mixed with and then applied to a sample grid without further treatment. Moreover, fibril samples obtained both with and without FN075 after fibrillation induced using a platereader were also investigated.

Grids were prepared as described by Smith and co-workers^30^. Briefly, 6 μL of the sample were loaded onto copper 400 mesh grids (Agar Scientific, Stansted, UK) coated with Formvar and carbon film. After 60 s, 10 μL of distilled water was added and excess water was removed. Subsequently, 10 μL of 2% (w/v) uranyl acetate (Agar Scientific) was placed on the grid and left for 30 s. Finally, 2 × 10 μL distilled water were added and again excess water was removed. The grid was then left to dry. Images were acquired at Core Facility for Integrated Microscopy (CFIM), at the Department of Health and Medical Sciences UCPH. Images were collected using a CM100 transmission electron microscope operating at an acceleration voltage of 80 kV.

### X-ray Fibre diffraction (FD)

Fibrils formed under four different experimental conditions were investigated: i) PBS, ii) PBS with FN075 corresponding to 1:1 molar ratio, iii) PBS with FN075 corresponding to 1:2 molar ratio and finally iv) PBS with DMSO corresponding to samples ii and iii. The protein concentration was 1 mg/ml, and the fibrils were prepared using the platereader as described above. Samples were extracted when the ThT curve had reached the plateau and were centrifugated at 18,000 rcf for 4 hours and left to dry in a desiccator. FD experiments were performed at the 911-2 MX beamline at Maxlab using a MAR165 CCD detector. Sample to detector distance was 220 mm and the wavelength used was 1.0385 Å (sample ii, exposure time 30 s) and 1.040 Å (samples i, iii and iv, exposure time 90 s). Cryostat was set to 4 °C and measurements were carried out in static mode, with the dried pellet mounted on a pin. Equatorial and meridional signals were plotted, by radially integrating 60° data about each respective axis using the *Radial Average* function of *CLEARER*^31^. Integrated diffraction signals were exported as a function of pixels and converted to the scattering angle in Matlab, using the pixel size and detector distance.

## Additional Information

**How to cite this article**: Nors Perdersen, M. *et al.* Direct Correlation Between Ligand-Induced α-Synuclein Oligomers and Amyloid-like Fibril Growth. *Sci. Rep.* **5**, 10422; doi: 10.1038/srep10422 (2015).

## Supplementary Material

Supplementary Information

## Figures and Tables

**Table 1:**
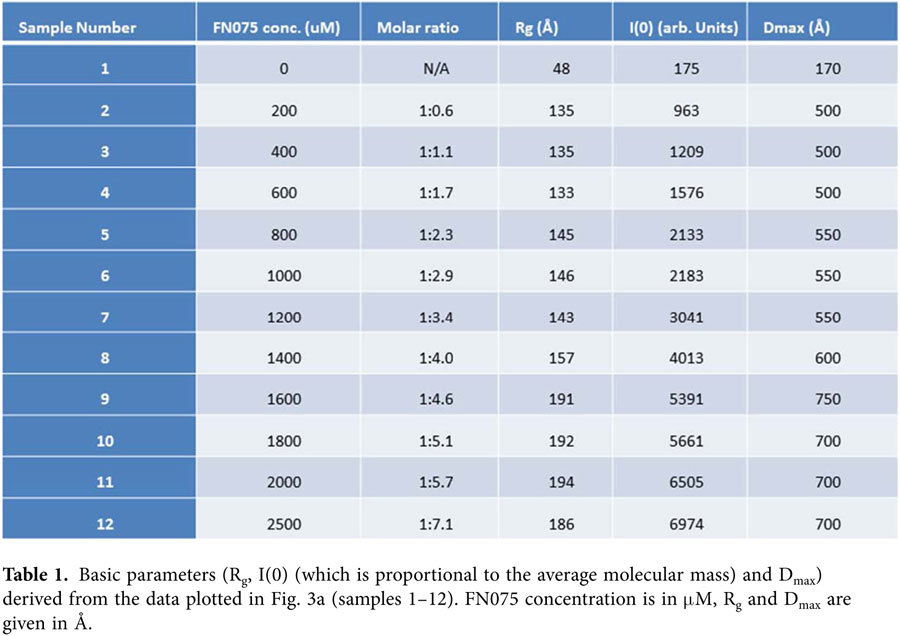
Basic parameters (R_g_, I(0) (which is proportional to the average molecular mass) and D_max_) derived from the data plotted in Fig. 3a (samples 1–12). FN075 concentration is in μM, R_g_ and D_max_ are given in Å.
