# Corrigendum: Direct Correlation Between Ligand-Induced α-Synuclein Oligomers and Amyloid-like Fibril Growth

**DOI:** 10.1038/srep15692

**Published:** 2015-10-28

**Authors:** Martin Nors Pedersen, Vito Foderà, Istvan Horvath, Andreas van Maarschalkerweerd, Katrine Nørgaard Toft, Christoph Weise, Fredrik Almqvist, Magnus Wolf-Watz, Pernilla Wittung-Stafshede, Bente Vestergaard

Scientific Reports 5: Article number: 1042210.1038/srep10422; published online: 05282015; updated: 10282015.

The original version of this Article contained a typographical error in the spelling of the author Martin Nors Pedersen, which was incorrectly given as Martin Nors Perdersen.

This has now been corrected in both the PDF and HTML versions of the Article.

