# Supplementary material for: Direct Correlation Between Ligand-Induced α-Synuclein Oligomers and Amyloid-like Fibril Growth
Source: Sci Rep. 2015 May 28;5:10422. doi: 10.1038/srep10422 (PMC4603703; doi:10.1038/srep10422)
Supplement: Supplementary Information [file srep10422-s1.pdf]

Direct Correlation Between Ligand-Induced α-Synuclein Oligomers and Amyloid-like Fibril Growth

Supplementary Information

Martin Nors Perdersen[1], Vito Foderà[1], Istvan Horvath[2], Andreas van Maarschalkerweerd[1], Katrine Nørgaard Toft[1], Christoph Weise[2], Fredrik Almqvist[2,3], Magnus Wolf-Watz[2], Pernilla Wittung-Stafshede[2,] and Bente Vestergaard[1]*

[1]Department of Drug Design and Pharmacology, University of Copenhagen Universitetsparken 2, DK-2100 Copenhagen, Denmark

[2]Department of Chemistry, Umeå University, S-901 87 Umeå, Sweden
[3]Umeå Centre for Microbial Research, Umeå University, 901 87 Umeå Sweden

Characterization of the structural conversion using nuclear magnetic resonance (NMR) – more detailed description.

Further insight into the process of oligomer formation was gained by recording a sequence of heteronuclear single quantum coherence (HSQC) spectra during incubation of freshly prepared aSN:FN075 samples. In the HSQC spectrum, the N-terminal and NAC-region cross-peaks attenuate during oligomerization, due to the slower tumbling of the large oligomers. Due to their flexibility, the surface exposed C-terminal residues are still observable, since they tumble faster than the remaining residues in the oligomer structure. The residue specific attenuation was investigated at four different protein:ligand ratios (Figure S1). The recorded spectra were then compared to a reference spectrum of native aSN. The ratio between the recorded spectrum and the reference is plotted in Figure S1. No significant attenuation of the signal is seen in any part of the spectrum with a ratio of 1:1.5 aSN:FN075, while, when the ratio is ~1:6, a strong attenuation of the N-terminal residues (blue color) is observed already within the first two hours of incubation. A clear pattern emerges when the summed amplitudes are plotted as a function of aSN:FN075 ratio as shown in Figure S1. Increased cross-peak attenuation is seen with increasing amount of FN075 and at a ratio around 1:6 an almost full attenuation is achieved for the N-terminal signal. Differing evolution of attenuation over time, or e.g. complete attenuation (either case corresponding to hetero-disperse oligomer formation), was not observed. As a consequence, the NMR data show that only one type of oligomer is formed under the experimental conditions investigated, irrespective of the aSN:FN075 ratio. The NMR kinetics also show that the oligomers, at the given protein concentration and for the time of investigation, appear stable. This is concluded since no further attenuation or changes in signal (neither intensity nor chemical shifts) is observed after the initial attenuation.[1]

Investigation of the cross sectional dimension of fibrils using TEM

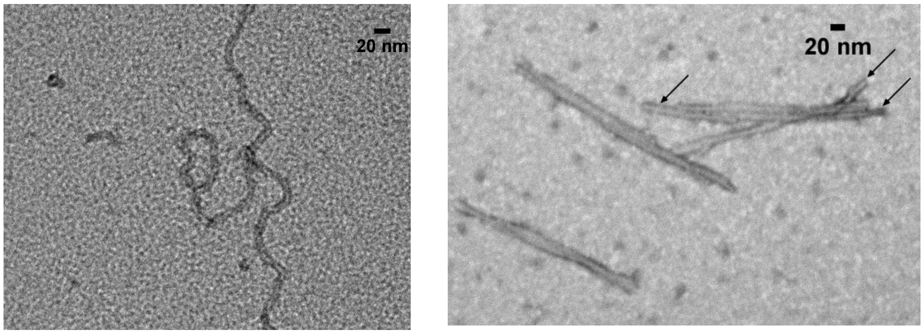

Figure S1: Two high magnification images of fibrils formed by sole addition of FN075 (left) and fibrils formed by platereader assay in presence of FN075 (right). The fibrils from the platereader have been fragmented by the glass beads and hence have a higher tendency to laterally associate. But the individual fibrils have the same cross section dimension (arrows in the figure point out single fibril strands)

Time evolution of aSN:FN075 samples

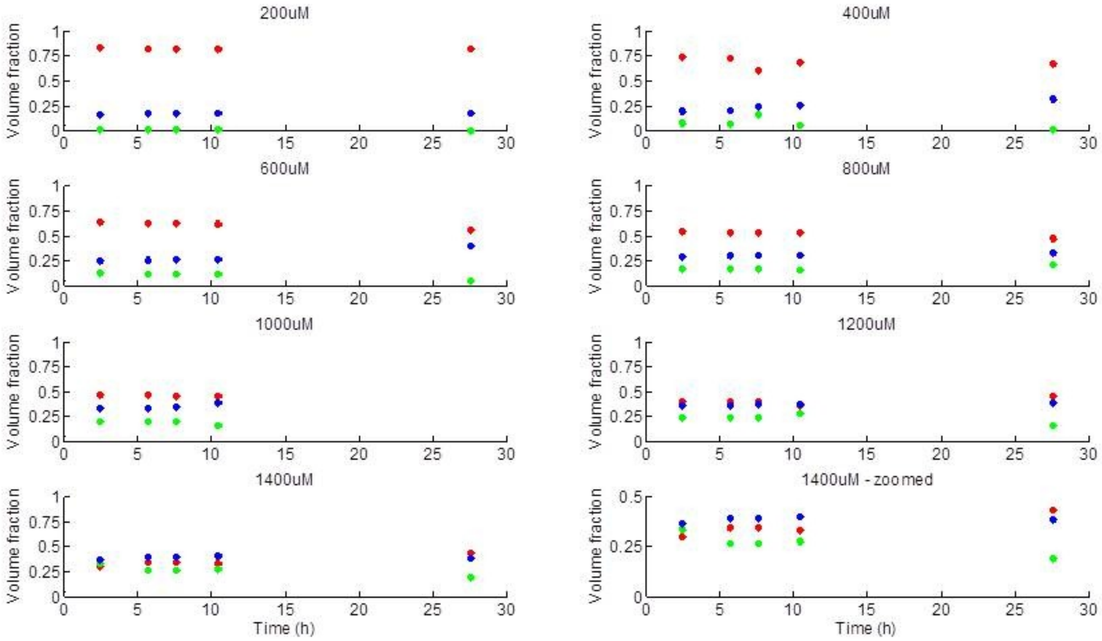

Figure S2: Time evolution of the first aSN:FN075 samples in the titration series. Volume fractions for monomers are red, those for oligomers are green and fibril volume fractions are blue. All plots are on the same scale except lower right corner, which is zoomed to highlight that the sample appeared contaminated.

Investigating the SAXS buffer scattering with and without FN075 present

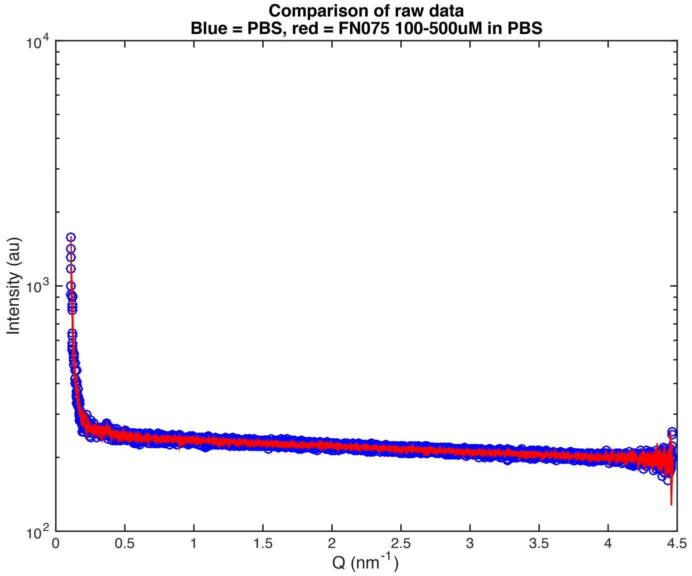

Figure S3: Comparison between the raw SAXS signal from the PBS-buffer and PBS with FN075. The blue empty circles correspond to the PBS measurements measured before and after each PBS+FN075 measurements, which all are plotted as red lines. Concentration range investigated is from 100-500 μM FN075. Above this concentration, FN075 phase separates.

Rod-guinier approximation:

	Fibrils w. FN075	Fibrils w/o FN075
Cross section I(0)	277	149
Cross section<br>Rg (Å)	66	67
Protomers per nm	1.6	0.85

Table S1: Parameters from the rod-guinier analysis of fibrils formed with and without FN075.

Supplementary Methods

SAXS data collection

Data were measured at the BioSAXS beamline P12 at Petra III, EMBL Hamburg using a momentum transfer (q)-range of 0.1095 nm-1 - 4.469 nm-1. Samples were loaded using the robotic setup[2, 3] and 20 individual exposures of 250 ms were recorded for each sample during flow. Individual frames from the measurements were inspected and frames revealing radiation damage were excluded from averaging by the pipeline[4]. Background was measured before and after measurement and averaged before being subtracted.

The fibril sample for the decomposition analysis was prepared in the platereader using 1 mg/ml aSN in PBS with 1:1 molar ratio of aSN:FN075. The sample was extracted when the ThT curve had reached a plateau. Fibril samples were measured at SAXS beamline 911-4 at Maxlab, Lund using a q-range of 0.0824 nm^{-1} to 5.58 nm^{-1}. Each sample was measured for 2 min and the sample was oscillating during measurement to avoid radiation damage. Background consisting of PBS with 20 μM ThT and DMSO was measured before and after each protein sample measurement. Data were corrected for detector response before being reduced in RAW[5]. Fibril samples for modeling the fibril repeating unit were prepared as above but measured at the BioSAXS beamline P12 at Petra III, EMBL Hamburg. The q-range for these measurements was 0.0488 nm^{-1} – 3.50 nm^{-1}.

In order to assure correct background subtraction, the buffer including FN075 in the concentration range of 0-500 μM FN075 was measured. No detectable difference was observed, see Figure S3. It was not possible to increase the concentration of FN075 above 500 μM since the molecule precipitates at higher concentrations. Only in the presence of protein, is it possible to dissolve higher concentrations of FN075. This is because FN075 binds to the protein oligomers, rather than existing in solution. From

this, it follows logically that the samples containing protein will have a maximum of 500 μM FN075 dissolved, hence our background check is sufficient at this point. However, also our NMR experiments confirm that FN075 is bound to the protein, rather than being free in solution (Figure S4). It is evident from Figure S4 that the signal for FN075 disappears in the presence of aSN. In conclusion, background subtraction using a buffer made from PBS with 20 μM ThT and DMSO is the most adequate solution.

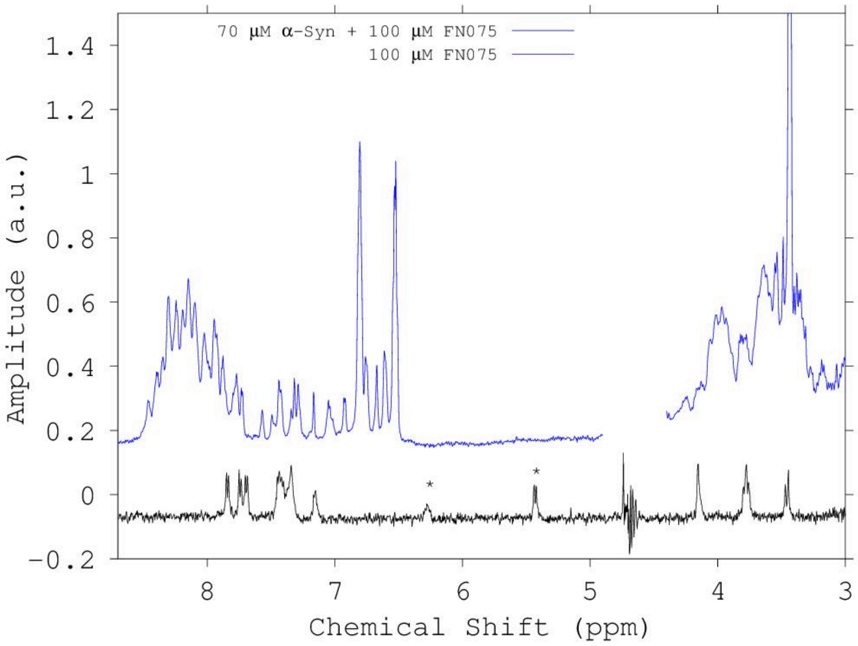

Figure S4: NMR spectra of 100 uM FN075 alone and 100 uM FN075 with 70 uM aSN. There are two peaks from FN075 that do not overlap with neither the spectrum from aSN alone or that of aSN with FN075. Both have been highlighted by asterisk. Notice that they are not present in the aSN with FN075 spectrum meaning that FN075 signal has been attenuated by binding to aSN.

It is evident from Figure S4 that the signal for FN075 disappears in the presence of aSN. In conclusion, background subtraction using a buffer made from PBS with 20 μM ThT and DMSO is the most adequate solution.

The *ab initio* modeling was performed by using the program DAMMIN[6]. Spherical search volume was used when modeling the oligomer and both spherical and cylindrical search volumes were used when modeling the fibrils. Data for the oligomer were truncated at 1.943 nm^{-1} and constant subtraction was enabled. Data for the fibril were truncated at 0.9992 nm^{-1} and constant subtraction was enabled. For each setting tested, 12 models were calculated. The ensembles of models were averaged analyzed using the DAMAVER suite[7]. The average of the models was used to create a new search volume, which was used for further refinement, by decreasing the bead size.

NMR:

^{1}H-^{15}N HSQC experiments were performed on a Bruker DRX600 NMR instrument using a cryoprobe fitted with z-axis gradients and pulse programs from the Bruker library. Samples for experiments consisted of 70 μM uniformly ^{15}N-labeled α-synuclein in 20 mM sodium phosphate pH 7.4 with 5-10% D_2O. The desired ratio of FN075 to protein was achieved by adding aliquots of a 100 mM solution of FN075 in DMSO. ^{1}H-^{15}N HSQC spectra were acquired using a so-fast pulse program variant with decoupling during acquisition. The 2D spectra were processed with nmrPipe[8] and resonance assignments were analyzed in Ansig for Windows[9].

References

1. Kwan AH, Mobli M, Gooley PR, King GF, Mackay JP. Macromolecular NMR spectroscopy for the non‐spectroscopist. *FEBS journal* **278**, 687-703 (2011).

2. Round A*, et al.* Automated sample-changing robot for solution scattering experiments at the EMBL Hamburg SAXS station X33. *J Appl Crystallogr* **41**, 913-917 (2008).

3. Blanchet CE*, et al.* Instrumental setup for high-throughput small-and wide-angle solution scattering at the X33 beamline of EMBL Hamburg. *Journal of Applied Crystallography* **45**, 489-495 (2012).

4. Franke D, Kikhney AG, Svergun DI. Automated acquisition and analysis of small angle X-ray scattering data. *Nuclear Instruments and Methods in Physics Research Section A: Accelerators, Spectrometers, Detectors and Associated Equipment* **689**, 52-59 (2012).

5. Nielsen S*, et al.* BioXTAS RAW, a software program for high-throughput automated small-angle X-ray scattering data reduction and preliminary analysis. *J Appl Crystallogr* **42**, 959-964 (2009).

6. Svergun D. Restoring low resolution structure of biological macromolecules from solution scattering using simulated annealing. *Biophys J* **76**, 2879-2886 (1999).

7. Volkov VV, Svergun DI. Uniqueness of ab initio shape determination in small-angle scattering. *Journal of Applied Crystallography* **36**, 860-864 (2003).

8. Delaglio F, Grzesiek S, Vuister GW, Zhu G, Pfeifer J, Bax A. NMRPipe: a multidimensional spectral processing system based on UNIX pipes. *Journal of biomolecular NMR* **6**, 277-293 (1995).

9. Helgstrand M, Kraulis P, Allard P, Härd T. Ansig for Windows: an interactive computer program for semiautomatic assignment of protein NMR spectra. *Journal of biomolecular NMR* **18**, 329-336 (2000).
